# Factors Influencing Continuance Intention for Online Consultations Among Survivors of Cancer: Grounded Theory Study

**DOI:** 10.2196/84644

**Published:** 2026-01-07

**Authors:** Yutang Yao, Musi Zhang, Shanshan Peng, Zhuzhong Cheng, Yun Duan

**Affiliations:** 1 Department of Nuclear Medicine Sichuan Cancer Hospital & Institute, Sichuan Cancer Center University of Electronic Science and Technology of China Chengdu, Sichuan China; 2 Department of Radiation Oncology Sichuan Cancer Hospital & Institute, Sichuan Cancer Center University of Electronic Science and Technology of China Chengdu, Sichuan China

**Keywords:** survivors of cancer, online consultation, telemedicine, digital health, grounded theory, continuance intention, patient engagement, physician-patient communication, privacy concerns, survivorship care

## Abstract

**Background:**

Online consultation platforms have become an important component of survivorship care for patients with cancer, offering flexible access to oncology expertise between scheduled visits. However, evidence on what drives the willingness of survivors of cancer to continue using online consultations after initial adoption remains limited in China. A better understanding of continuance intention is needed to inform survivor-centered digital health strategies.

**Objective:**

This study aimed to explore the influencing factors of continued use of online consultations among survivors of cancer in southwest China and develop a grounded theoretical model explaining continuance intention.

**Methods:**

A grounded theory qualitative design was used. A total of 26 adult survivors of cancer with diverse demographic and clinical characteristics were purposively recruited from a tertiary cancer center in southwest China. All participants had used online consultations at least once in the preceding year. Semistructured telephone interviews were audio recorded; transcribed verbatim; and analyzed using open, axial, and selective coding with constant comparison until theoretical saturation was reached. During selective coding, categories and their relationships were integrated and iteratively refined to construct a grounded theoretical model of continuance intention.

**Results:**

Six interrelated domains influenced survivors’ continued use of online consultation platforms: platform quality, physician competence, user perception, individual condition, external context, and privacy concerns. Platform quality and physician competence influenced user perception of usefulness, reassurance, and trust, which functioned as a mediator of continued use. Individual condition, including health status, health literacy, and psychological needs, influenced both perceived usefulness and reliance on online consultations. External context, especially family encouragement, peer recommendations, and availability of local oncology services, directly facilitated or constrained continued use. Privacy concerns moderated how survivors balanced perceived benefits against risks of data misuse, stigma, and unwanted disclosure of cancer history. Survivors described online consultations as offering rapid guidance and emotional support that complemented hospital-based care but reported discontinuation when interactions were delayed or impersonal or when perceived privacy risks outweighed the benefits.

**Conclusions:**

The willingness of survivors of cancer to continue using online consultation platforms depends on multiple interrelated factors beyond traditional technological usability. Sustained engagement is shaped by survivors’ perceptions of usefulness and trust, physician empathy and timeliness, family encouragement, and acceptance of privacy trade-offs. The theoretical model advances understanding of digital health continuance in oncology and offers practical guidance for developing survivor-centered online consultation services.

## Introduction

### Background

Cancer remains one of the leading causes of morbidity and mortality worldwide, placing a tremendous burden on health systems, families, and individuals. Advances in diagnosis and treatment have improved survival rates, and as a result, the population of survivors of cancer continues to expand [1-3]. However, survivorship is not simply the period after treatment. It also includes ongoing challenges such as long-term side effects, fear of recurrence, psychosocial adjustment, and the need for continuous follow-up care. The World Health Organization and many national cancer control programs have emphasized the importance of survivorship care as an integral component of comprehensive cancer management [4,5].

Digital health technologies have become increasingly important in bridging gaps in access to care, especially for patients requiring long-term monitoring [6-8]. Online consultation platforms, accessed through mobile apps or web portals, allow survivors to communicate with oncology professionals, seek advice on symptoms or lifestyle modifications, and receive psychological support. For survivors living in regions with limited oncology resources, online consultation provides a vital link to specialists otherwise geographically inaccessible [9-12]. Even in urban centers with advanced hospitals, the platforms offer convenience and continuity between scheduled in-person visits [13,14].

The COVID-19 pandemic further accelerated the adoption of telemedicine and online health services, demonstrating their potential to supplement traditional care [14-16]. Survivors of cancer, often immunocompromised, benefitted from reduced exposure to hospital environments through digital consultations. As health systems adapt to postpandemic realities, online consultation is positioned as a permanent component of integrated cancer survivorship care. However, the long-term viability of such services depends not merely on initial adoption but also on survivors’ willingness to continue using them. Therefore, understanding the factors that shape continued use is essential for sustaining the role of online consultation in oncology care.

Research on online consultation and telemedicine has expanded rapidly in recent years, with a growing focus on use patterns and determinants of adoption [17-19]. Several theoretical models have guided this scholarship. The technology acceptance model (TAM) and expectation confirmation model (ECM) are frequently used to examine perceived usefulness, ease of use, and satisfaction as drivers of continued use. Studies across various digital health contexts, including mobile health apps, wearable devices, and patient portals, have confirmed that perceived usefulness and trust are strong predictors of sustained engagement [19-22].

In oncology, early work has highlighted the potential of online consultation to support symptom management, medication adherence, and communication between patients and health care professionals. For example, randomized controlled trials involving survivors of breast cancer have demonstrated that digital follow-up systems improve quality of life and reduce hospital visits [23-26]. Observational studies in Europe and Asia have reported that online consultation platforms are especially effective in providing dietary advice, managing side effects, and delivering psychosocial support to survivors [27-29].

A growing body of literature has investigated continuance intention in digital health contexts. In nononcology populations, continuance is influenced by habit formation, social influence, and perceived quality of service. For instance, research on chronic disease management apps has found that individuals with strong digital literacy and positive reinforcement from peers are more likely to continue use [30-32]. Other studies have identified cost, accessibility, and integration with offline care as determinants of sustained use [33,34]. However, in cancer populations, empirical evidence remains fragmented. Some studies of survivors of breast cancer have revealed that online consultation provides emotional reassurance and complements offline care [35-37], whereas others have found that survivors discontinue use due to inconsistent physician availability and lack of trust [38].

In this study, continuance intention refers to survivors’ intention to keep using an online consultation platform after initial adoption rather than a single episode of use. In digital health, continuance intention is essential because the benefits of telemedicine and online consultation usually accumulate through repeated contacts, ongoing symptom management, and sustained relationships with clinicians. If survivors discontinue use after early trials, online services may show good initial uptake yet fail to deliver long-term gains in symptom control, psychological support, or care coordination.

While prior studies provide valuable insights into adoption of and satisfaction with online consultations, few have systematically investigated the factors influencing continued use by survivors of cancer over time. Most quantitative research has relied on preexisting models such as the TAM or ECM, which capture perceptions of technology but may not adequately reflect the lived realities of survivorship. Survivors navigate a complex interplay of medical, psychological, social, and technological factors. Their willingness to sustain engagement with online consultation platforms cannot be reduced to perceived usefulness alone.

There is also a lack of theory-building research specific to oncology survivorship in the Chinese context. Most studies of digital health continuance have applied established frameworks such as the TAM and ECM with a focus on perceived usefulness, ease of use, and satisfaction. Such models were not developed for the complex realities of cancer survivorship, where survivors simultaneously manage late effects, fear of recurrence, family expectations, and resource constraints in the health system. Without theory grounded in survivors’ lived experiences, especially within Chinese sociocultural and institutional settings, it is difficult to design online consultation services that are both acceptable and sustainable. Theory-building work can clarify which technological, relational, and contextual influences matter most for continuance and how they interact during survivorship care.

### Objectives

Therefore, this study aimed to explore the factors that influence the continued use of online consultation platforms by survivors of cancer in southwest China and develop a grounded theoretical model explaining continuance intention in this context. Specifically, this study sought to identify individual, relational, and contextual influences on continued use and articulate how such influences interrelate within survivorship care.

## Methods

### Study Design

This study used a grounded theory qualitative design to examine factors influencing continuance intention regarding online consultations among survivors of cancer. The approach by Strauss and Corbin [39] was adopted, with iterative cycles of data collection and analysis and a 3-stage process of open coding, axial coding, and selective coding [40]. Constant comparative analysis was used to compare incidents within and across interviews and refine categories and their properties as the theoretical model developed [41].

### Study Setting

This study was conducted at the Sichuan Cancer Hospital and Institute, Sichuan Cancer Center, the Affiliated Cancer Hospital of the University of Electronic Science and Technology of China. This institution is the largest national tertiary cancer hospital in southwest China, with comprehensive functions in cancer prevention, treatment, rehabilitation, research, and education. Its extensive clinical services and diverse patient population made it an ideal setting for examining the experiences of survivors of cancer with online consultation platforms.

### Participants and Recruitment

Participants were adult survivors of cancer with diverse cancer types and in diverse survivorship stages who had used an online consultation platform at least once in the preceding year. Inclusion criteria were age of ≥18 years, confirmed cancer diagnosis, completion of initial treatment or in active follow-up, at least one prior online consultation related to cancer care within the previous year, and ability to communicate via telephone in Mandarin. Survivors with severe cognitive impairment or acute clinical distress or who were unable to communicate effectively via phone were excluded. The criterion of at least one recent online consultation ensured that participants could describe concrete experiences with platform use and decisions about continued use rather than hypothetical views.

Recruitment followed purposive sampling to maximize diversity in age, gender, cancer type, treatment stage, and place of residence. During outpatient follow-up appointments, clinical nurses and oncologists briefly introduced the study to eligible survivors and, with permission, shared contact details with the research team. The team then telephoned interested survivors to provide detailed study information, confirm eligibility, and arrange an interview time. In survivorship support groups and patient-led online forums, a short study notice invited interested survivors to contact the team directly. All invitations were active rather than open public advertisements. Degree of experience with online consultation and digital literacy were not used as formal sampling strata. Instead, the focus was on variation in survivorship trajectories and clinical backgrounds. Digital literacy was not assessed using a standardized scale, which is acknowledged as a limitation when interpreting differences in continuance intention.

### Data Collection

Data were collected through semistructured telephone interviews, which were chosen to accommodate survivors living in different regions and reduce travel burden. After eligibility was confirmed, interviews were scheduled at times convenient for participants, usually outside routine clinic visits. Two trained oncology nurses (YY and MZ) conducted all interviews. Both interviewers worked at the same tertiary cancer center but were not part of the clinical team directly responsible for participants’ current treatment. At the start of each interview, the interviewer introduced their professional background, clarified the voluntary nature of participation, and emphasized that decisions about care would not be affected by participation. Interviews followed a flexible guide covering experiences with online consultations, reasons for continued use or discontinuation, family and social influences, and privacy concerns. (Multimedia Appendix 1) Interviews lasted between 25 and 40 minutes, were audio recorded with permission, and were transcribed verbatim in Mandarin. Each transcript was anonymized and assigned an identifier (C01 to C26).

### Data Analysis

Data analysis began after the first interviews and proceeded concurrently with ongoing data collection. Two researchers (YY and MZ) conducted line-by-line open coding on an initial set of transcripts to identify concepts related to survivors’ experiences of online consultations, perceived benefits and drawbacks, relational and family influences, and privacy concerns. (Multimedia Appendix 2) Codes were compared, merged, and refined in regular meetings, and a preliminary coding framework was developed.

During axial coding, conceptually similar codes were clustered into categories, and relationships among categories were explored using constant comparison across participants and time points. Analytic memos documented emerging ideas about potential mediators, moderators, and contextual conditions influencing continuance intention. In the selective coding stage, categories were integrated into 6 higher-level domains and a core category of continuance intention. The developing theoretical model was iteratively checked against the data, including accounts that appeared to deviate from early interpretations, and revised until it accounted for the range of observed patterns.

Coding was conducted independently by the 2 researchers, and discrepancies were discussed and resolved through consensus, with a third researcher (YD) consulted when needed. An audit trail including codebooks, memos, and diagrams was maintained to support transparency and dependability. Sampling became increasingly focused as analysis progressed, for example, by recruiting survivors from different age groups and residential areas once the importance of family involvement and privacy concerns became clear. Theoretical saturation was assessed after 23 interviews when no new categories were identified and relationships between domains appeared stable. Three additional interviews were conducted to confirm saturation and ensure that the model held true for more recent cases.

### Rigor and Trustworthiness

Credibility and trustworthiness were supported through multiple strategies. First, purposive and iterative sampling captured survivors with diverse demographic and clinical backgrounds to enhance variation in experiences. Second, 2 researchers independently coded transcripts and compared interpretations in regular analysis meetings, with disagreements resolved through discussion and involvement of a third researcher where required. Third, constant comparison and negative case analysis were used to test whether the evolving model could accommodate accounts that challenged early assumptions. Fourth, an audit trail of coding decisions, memos, and diagrams was maintained to support dependability and confirmability. Finally, brief member checking was conducted with 4 participants who were invited to comment on thematic summaries; they confirmed that the domains and relationships reflected their experiences. Reporting follows the Standards for Reporting Qualitative Research and is informed by the COREQ (Consolidated Criteria for Reporting Qualitative Research) guidelines.

### Researcher Positionality

The research team consisted of oncology clinicians and nursing researchers working in a tertiary cancer center in southwest China. The interviewers were oncology nurses with long-standing experience caring for survivors during treatment and follow-up, which facilitated rapport but may also have shaped the topics explored and the way in which participants described their care. The senior author is a nuclear medicine physician with experience in survivorship care and digital health initiatives in the hospital where this study was conducted. The team acknowledges that familiarity with hospital-affiliated online platforms and a generally positive view of digital health could influence interpretation of the data. To address this, assumptions were documented in analytic memos, and team discussions explicitly considered alternative explanations and accounts that did not align with expectations.

### Ethical Considerations

The study protocol was reviewed and approved by the ethics committee of Sichuan Cancer Hospital and Institute (approval SCCHEC-02-2020-036). All participants received verbal and written information about the study and provided informed verbal consent before the interviews. Participation was voluntary, and survivors could decline questions or withdraw at any time without consequences for their clinical care.

To protect privacy and confidentiality, audio recordings were stored on password-protected devices accessible only to the research team, and transcripts were deidentified by removing names and other direct identifiers. Potentially identifying combinations of demographic and clinical details were aggregated in reporting so that individual participants could not be recognized. No financial incentives or material compensation were provided for participation.

## Results

### Participant Characteristics

The 26 participants included 15 (58%) women and 11 (42%) men, with most being middle-aged (40-49 years: n=8, 31%; 50-59 years: n=7, 27%). Survivors of breast cancer constituted the largest group (n=7, 27%), followed by lung (n=4, 15%) and colorectal (n=3, 12%) cancer, whereas other cancer types such as cervical, ovarian, prostate, gastric, and thyroid cancer each represented 8% (n=2), and liver and kidney cancer each represented 4% (n=1). Survivorship stages were balanced, with 31% (n=8) in active treatment and 35% (n=9) each in remission and long-term survivorship. Educational levels ranged from 23% (n=6) with a high school or lower level to 12% (n=3) with postgraduate education. Most participants (n=11, 42%) were employed, and 62% (n=16) lived in urban areas as presented in Table 1.

Analysis identified 6 domains shaping the continued use by survivors of cancer of online consultation platforms: platform quality, physician competence, user perception, individual condition, external context, and privacy concerns. The interrelationships among the domains formed a theoretical model explaining continuance intention.

**Table 1:** Sociodemographic and clinical characteristics of survivors of cancer participating in grounded theory interviews on continuance intention regarding online consultations in southwest China (N=26).

Category		Participants, n (%)
**Gender**		
	Female	15 (58)
	Male	11 (42)
**Age (years)**		
	30-39	5 (19)
	40-49	8 (31)
	50-59	7 (27)
	≥60	6 (23)
**Cancer type**		
	Breast	7 (27)
	Lung	4 (15)
	Colorectal	3 (12)
	Cervical	2 (8)
	Ovarian	2 (8)
	Prostate	2 (8)
	Gastric	2 (8)
	Thyroid	2 (8)
	Liver	1 (4)
	Kidney	1 (4)
**Treatment stage**		
	Active treatment	8 (31)
	Remission	9 (35)
	Long-term survivorship	9 (35)
**Educational level**		
	High school or lower	6 (23)
	College or junior college	10 (38)
	Bachelor’s degree	7 (27)
	Master’s degree or higher	3 (12)
**Occupation status**		
	Employed	11 (42)
	Retired	7 (27)
	Homemaker	3 (12)
	Self-employed	3 (12)
	Unemployed	2 (8)
**Geographic location**		
	Urban	16 (62)
	Semiurban or rural	10 (38)

### Platform Quality

Platform quality encompassed service quality and information quality. Survivors valued consistent access to oncology specialists, transparent consultation fees, reliable technical support, and effective complaint resolution. Information quality included completeness of physician profiles, clarity of treatment explanations, and access to trustworthy cancer-related educational content as presented in Table 2.

**Table 2:** Themes and subthemes related to platform quality influencing continuance intention regarding online consultations among survivors of cancer.

Subtheme	Key insights	Quote
Service quality	Survivors expected efficient systems, reasonable fees, and transparent policies.	“The charges were clear. I could reach the same oncologist again. The convenience made me continue.” [C04; male; survivor of gastric cancer]
Information quality	Accurate physician and treatment information increased trust.	“I can see which doctors are experts or which are younger doctors. It’s easier than going to the hospital.” [C21; female; survivor of ovarian cancer]

### Physician Competence

Physician competence emerged as central. Survivors highlighted professional expertise, communication ability, and timeliness. Expertise reassured patients on side effect management and recurrence risks. Compassionate communication provided emotional support. Timely responses were critical, especially during treatment as presented in Table 3.

**Table 3:** Themes and subthemes related to physician competence influencing continuance intention regarding online consultations among survivors of cancer.

Subtheme	Key insights	Quote
Professional expertise	Detailed, tailored explanations increased trust.	“When the doctor explained why my fatigue persisted, it felt like guidance I could trust.” [C18; male; survivor of lung cancer]
Communication ability	Survivors valued empathy and encouragement.	“She told me that anxiety was normal, and suddenly I felt less alone. That made me come back.” [C09; female; survivor of breast cancer]
Timeliness	Delays discouraged use; quick replies reinforced reliance.	“During chemo, hours felt like days. A fast reply was the reason I kept using the app.” [C23; male; survivor of colorectal cancer]

### User Perception

User perception comprised ease of use, perceived usefulness, and positive expectation. Survivors emphasized the importance of intuitive interfaces and smooth navigation. Usefulness was defined as reassurance between hospital visits, management of treatment side effects, and guidance for lifestyle adaptation. Positive expectation referred to trust in the future development of digital health services.

Survivors described online consultations as a lifeline during uncertain recovery phases. One participant noted the following:

Even when nothing urgent, knowing I could reach professional doctors quickly gave me comfort.

C06; female; survivor of cervical cancer

### Individual Condition

Individual condition referred to health literacy, health status, and personal needs. Survivors with higher health literacy were more confident in engaging online, whereas those with limited literacy faced challenges in sustained use. Health status influenced patterns: those experiencing lingering treatment effects frequently sought reassurance, whereas long-term survivors without active symptoms used consultation more sporadically. Needs extended beyond medical queries to psychological reassurance and lifestyle advice.

One participant said the following:

After surgery I still had numbness in my hands, and I didn’t always know if it was normal. Having the online consultation gave me a way to check quickly without waiting weeks for my hospital appointment.

C02; female; aged 52 years; survivor of breast cancer

### External Context

External context included family encouragement, peer influence, and offline health care alternatives. Family members often facilitated continued use, particularly adult children assisting older survivors with technology. Peer groups shared recommendations of reliable platforms, which reinforced trust. Survivors in rural areas with limited oncology services relied heavily on online consultations, whereas those living near tertiary hospitals sometimes preferred in-person visits.

For instance, a participant mentioned the following:

My son insisted I keep using the app. He said it was safer than traveling two hours to the hospital every time I worried about something small, and he even helped me learn how to pay for consultations.

C09; male; aged 63 years; survivor of colorectal cancer

### Privacy Concerns

Privacy concerns centered on disclosure of medical records, genetic test results, and sensitive images. Survivors expressed anxiety about misuse of their cancer history but balanced this against perceived benefits. When consultations provided timely reassurance, survivors accepted privacy trade-offs.

One survivor explained the following:

I hesitated before uploading my CT scans, but the doctor’s advice was worth it.

C12; male; survivor of prostate cancer

### Interrelationships Among Domains

The findings revealed that platform quality and physician competence had a strong influence on user perception of online consultations, including perceived usefulness, reassurance, and trust. User perception, in turn, mediated the relationship between these domains and continuance intention. Survivors’ decisions to continue or discontinue online consultations depended on how they interpreted their cumulative experiences rather than on isolated platform attributes. Individual condition, including health status, health literacy, and psychological needs, directly shaped both perceived usefulness and reliance on online consultations. External context, such as family encouragement, peer influence, and accessibility of local oncology services, provided structural conditions that either facilitated or constrained continued use. Privacy concerns moderated the influence of other domains by intensifying or weakening the impact of perceived benefits. Survivors with high privacy concerns sometimes restricted or stopped use despite recognizing benefits, whereas institutional trust in hospital-affiliated platforms could soften privacy fears and support continued engagement (Table 4).

**Table 4:** Representative relationships among domains in the grounded theory model and their descriptions.

Relationship	Description
User perception→intention	Perceptions of usefulness and trust shaped continuity.
Platform quality→perception	Service quality enhanced trust and ease of use.
Physician competence→perception	Physician expertise built reassurance.
External context→intention	Peer recommendations influenced adoption.
Individual condition→intention	Active symptoms increased reliance.
Privacy concern→intention	Anxiety reduced willingness.

### Theoretical Model

**Figure 1:**
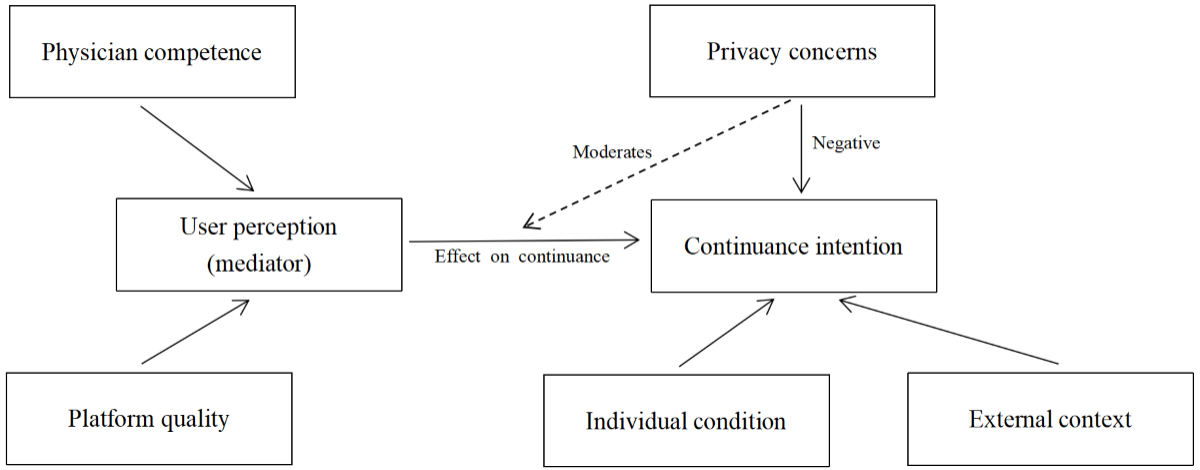
Grounded theory model of continuance intention for online consultations among survivors of cancer. Platform quality and physician competence influence continuance intention indirectly through user perception (mediator). Individual condition and external context have direct effects on continuance intention. Privacy concerns have a direct negative effect on continuance intention and moderate the association between user perception and continuance intention (N=26).

The final theoretical model positions continuance intention at the center, influenced by 6 interconnected domains. Platform quality and physician competence directly influence user perception, which mediates the relationship with continuance. Individual condition and external context exert both direct and indirect effects. Privacy concerns act as a contextual moderator, influencing the strength of survivors’ willingness to continue (Figure 1).

## Discussion

### Principal Findings

Analysis of in-depth interviews with 26 Chinese survivors of cancer identified 6 interrelated domains that shaped continuance intention: platform quality, physician competence, user perception, individual condition, external context, and privacy concerns. The domains were integrated into a theoretical model illustrating how survivors’ decisions to continue using online consultation platforms were mediated by perceptions of usefulness, trust, and accessibility while being simultaneously shaped by their personal health status, family influences, and privacy considerations. The findings underscore that continued use is not determined solely by technological ease or usefulness but emerges from a dynamic negotiation among survivors’ individual needs, relational contexts, and systemic constraints.

### Comparison With Prior Work

Previous studies have consistently identified perceived usefulness, trust, and habit formation as determinants of sustained use. For example, research on mobile disease management apps has demonstrated that convenience and ease of monitoring reinforce long-term engagement [42,43]. Other studies have linked continuance intention to perceptions of quality of physician feedback and integration into daily routines [44,45]. This suggests that the mechanisms of continuance identified in our study are not unique to oncology but resonate with broader digital health behaviors.

Survivors of cancer often experience uncertainty long after completion of primary treatment, with concerns about recurrence, lingering side effects, and psychosocial adjustment [46-48]. Unlike users of general wellness or chronic disease apps, survivors in our study emphasized the importance of empathetic physician communication and reassurance as key reasons for continuing online consultations. This aligns with studies on survivors of breast cancer, which have found that online consultations offer emotional support and alleviate isolation [49,50]. By centering survivors’ voices, our grounded theory extends beyond existing quantitative models that have often prioritized technological features over relational care.

Privacy concerns emerged as a moderator of continuance intention. Prior work has documented anxiety about data security in telemedicine broadly [51,52], but our findings reveal that survivors actively weigh the risks of disclosure against perceived benefits of reassurance. For example, some participants expressed hesitation about uploading genetic test results or sensitive images but, ultimately, continued use when the consultation reduced uncertainty. This dynamic risk-benefit trade-off is less discussed in the literature but resonates with recent studies in Chinese mobile health contexts, which report that patients are more willing to share personal data when platforms are affiliated with reputable hospitals [53]. Our study shows that this negotiation is particularly salient in oncology, where personal health information carries social and familial implications.

The findings need to be interpreted within the sociocultural context of southwest China. Survivors’ decisions about online consultations were often negotiated within families rather than made individually. Adult children in particular encouraged continued use, assisted with technical tasks, and sometimes controlled access to online services. Trust in public hospital-affiliated platforms reduced worries about data misuse compared with commercial platforms and supported acceptance of privacy trade-offs. At the same time, differences in digital literacy and internet access between urban and rural areas and between younger and older survivors shaped how readily survivors could use and benefit from online consultations. Such features of the Chinese context influence the transferability of the model to other settings and illustrate the value of theory development that is grounded in local sociocultural dynamics.

The configuration of the model would likely differ in health care contexts characterized by lower institutional trust or more individualistic decision-making. In low-trust settings, institutional affiliation may be less able to buffer privacy concerns, and privacy concerns may exert a more direct negative effect on continuance rather than primarily moderating perceived benefits. In more individualistic contexts, the external context pathway via family facilitation may be weaker, whereas individual appraisal of risk, autonomy, and personal preference may play a stronger role in shaping continuance intention.

### Theoretical and Practical Implications

This study advances understanding of digital oncology care in several ways. It focused on continuance intention rather than on initial adoption or satisfaction, emphasizing the long-term dynamics of survivor engagement. It developed a theory-informed framework grounded in the lived experiences of survivors of cancer in China, addressing an important gap in survivorship research. The model highlights how technological features, physician competence, survivor perceptions, individual conditions, family and social contexts, and privacy concerns interact to shape continued use of online consultation platforms. As a result, it offers a more comprehensive account of digital health continuance and practical guidance for policymakers, health care institutions, and technology developers who seek to design sustainable services for survivors of cancer.

Theoretically, this study shows the value of grounded theory for examining continuance intention in digital health and extends work based on the TAM and the ECM. Existing frameworks foreground technological usability, perceived usefulness, and satisfaction. In contrast, this model integrates relational, contextual, and personal health factors as core elements of continuance, thereby expanding the conceptual vocabulary for studying sustained engagement with health technologies. Unlike consumer technologies where continuance is often driven by convenience, entertainment, or routine fit, continuance intention in oncology survivorship is structurally anchored in clinical uncertainty, perceived vulnerability, and reliance on professional reassurance between episodic in-person visits. Survivors re-engage when symptoms, late effects, or fear of recurrence create a need for interpretation and emotional stabilization, making the relationship with clinicians and the perceived safety of the channel central to sustained use. In this context, reassurance and trust operate as distinct relational mechanisms rather than as subcomponents of perceived usefulness. Reassurance reflects reduction of uncertainty and emotional distress through clinician responsiveness and empathy, whereas trust reflects confidence in physician competence and institutional credibility. Both shape whether survivors interpret online consultations as safe and legitimate for ongoing survivorship management, which explains why user perception mediates continuance intention through reassurance and trust in addition to instrumental usefulness.

The model also clarifies the role of user perception. Survivors’ willingness to continue was shaped not only by platform quality or physician competence in isolation but also by how combined experiences influenced perceptions of usefulness, trust, and reassurance. User perception functioned as an active interpretive process that mediated the impact of system attributes and survivorship trajectories on continuance intention. In addition, this study offers a more refined understanding of privacy concerns in digital health. Privacy concerns operated as a dynamic moderator that affected the strength of continuance intention depending on how survivors weighed perceived benefits against perceived risks. This view moves beyond simple classifications of users as either concerned or unconcerned and encourages consideration of continuance as an ongoing negotiation.

From a practical standpoint, the findings suggest several priorities for online consultation services in oncology. Platforms should ensure clear physician profiles, transparent fee structures, and reliable technical support so that survivors can form stable expectations about service quality. Clinicians who provide online consultations require support and training in empathetic communication to address survivors’ psychological and emotional needs in addition to clinical questions. Timely responses are particularly critical for survivors in active treatment, which supports the integration of triage protocols, clear coverage arrangements, and notification systems that reduce avoidable delays. Addressing privacy concerns calls for transparent communication about data protection policies and visible affiliation with trusted institutions; survivors in this study expressed greater confidence in platforms linked to tertiary hospitals. Finally, given the central role of family encouragement, platform features that facilitate caregiver involvement, such as options for shared access or family-oriented consultation modes, may align with prevailing cultural practices and help sustain survivor engagement over time.

### Limitations

First, this study was conducted in a single regional tertiary cancer center in southwest China. Although the institution serves a large and diverse catchment area, experiences in other regions or health systems may differ. Second, the sample comprised survivors who were willing and able to participate in telephone interviews and who had had at least one prior online consultation, which may bias findings toward survivors who are more engaged with digital services. Standardized measures of digital literacy and prior telehealth experience were not collected, limiting the ability to quantify their influence on continuance intention. Third, interviews were conducted by oncology nurses affiliated with the same institution, which may have introduced social desirability bias or inhibited criticism of hospital-based platforms despite efforts to emphasize independence from clinical decisions. Finally, qualitative findings are interpretive and context specific; future research using mixed methods and larger samples is needed to test and refine the model in other oncological and cultural settings.

### Future Directions

Future research should test the theoretical model developed in this study through quantitative methods. Large-scale surveys could examine the relative influence of the 6 domains and validate their interrelationships. Cross-cultural comparative studies would be valuable to assess whether the role of family support and privacy negotiation is unique to China or generalizable to other contexts. Furthermore, longitudinal research could explore how continuance intention evolves across different phases of survivorship, from active treatment to long-term follow-up.

Intervention studies could also evaluate strategies to strengthen continuance. For instance, training programs for physicians in digital empathy or platform designs that allow for caregiver participation may enhance perceptions of usefulness and trust. Policy research should investigate frameworks for data protection that balance survivors’ privacy with the need for effective digital oncology services. As cancer survivorship continues to grow worldwide, such research is critical for integrating online consultations into sustainable, patient-centered models of care.

### Conclusions

Six interrelated domains influenced the continuance intention of survivors of cancer to use online consultation platforms: platform quality, physician competence, user perception, individual condition, external context, and privacy concerns. The grounded theory model shows that continued use results from a dynamic negotiation between perceived benefits and perceived risks that is shaped by survivors’ health needs, family roles, and institutional trust. Beyond summarizing determinants of service use, the model provides a theory-based foundation for designing and evaluating online consultation services that are more responsive to survivors’ long-term needs. By clarifying how technological, relational, and contextual influences interact in a Chinese oncology setting, this study contributes to broader efforts to build sustainable and equitable digital health systems in cancer survivorship care.

## Appendix

Multimedia Appendix 1 Semistructured interview guide.

Multimedia Appendix 2 Open coding concepts.

## Abbreviations

COREQ: Consolidated Criteria for Reporting Qualitative Research
ECM: expectation confirmation model
TAM: technology acceptance model

## Data Availability

The datasets generated or analyzed during this study are available from the corresponding author on reasonable request.
