# Supplementary material for: Factors Influencing Continuance Intention for Online Consultations Among Survivors of Cancer: Grounded Theory Study
Source: J Med Internet Res. 2026 Jan 7;28:e84644. doi: 10.2196/84644 (PMC12824571; doi:10.2196/84644)
Supplement: Multimedia Appendix 1 [file jmir_v28i1e84644_app1.pdf]

Appendix 1. Semi-structured interview guide

Introduction script (for interviewer to read)

Thank you for participating in this study. We want to understand your experiences with online consultation after cancer treatment. The goal is to learn how you use these services, what you think about them, and what makes you continue or stop using them. There are no right or wrong answers. Please share as much detail as you feel comfortable with. The interview will last about 25–40 minutes. With your permission, I will record our conversation to ensure accuracy. All information will be kept confidential and anonymized.

Section 1. General experience

1. Can you tell me about your journey with cancer and where you are now in your treatment or recovery?
2. How did you first hear about online consultation services for medical care?
3. Can you describe your first experience using online consultation?

Section 2. Patterns of use

4. How often do you use online consultation services?
5. In what kinds of situations do you usually decide to use them?
6. Have there been times when you chose not to use them? Why?

Section 3. Experiences with online consultation

7. What do you usually look for when you consult online?
8. Can you describe a time when online consultation was especially helpful to you?
9. Have you ever had a disappointing or negative experience with online consultation? What happened?
10. How do you feel about the difference between consulting online and visiting the hospital in person?

Section 4. Perceptions and feelings

11. What do you think are the main advantages of online consultation for someone in your situation?
12. What do you think are the main disadvantages?
13. How do you feel when you use online consultation — for example, about convenience, trust, or safety?

Section 5. Influences and support

14. Has anyone encouraged you to use online consultation, such as family members, friends, or doctors?

15. Have you discussed your experiences with other survivors or patient groups? If so, what did they say?

Section 6. Privacy and information sharing

16. How do you feel about sharing your personal health information or medical records online?
17. Have you ever felt worried about privacy or security when using online consultation?

Section 7. Continuation and future perspectives

18. Do you think you will keep using online consultation in the future? Why or why not?
19. What would make you more likely to continue using these services?
20. If you could change or improve anything about online consultation, what would it be?

Closing Script

Thank you for taking the time to talk with me. Your experiences are very valuable and will help us better understand how cancer survivors use online consultation services.
